# Vague Presentation of Cat Scratch Disease in a Child

**DOI:** 10.31486/toj.23.0086

**Published:** 2024

**Authors:** Alexander Crowley, Bhumit Desai, Sean Waldron

**Affiliations:** ^1^ Department of Orthopedic Surgery, Ochsner Clinic Foundation, New Orleans, LA; ^2^ The University of Queensland Medical School, Ochsner Clinical School, New Orleans, LA

**Keywords:** Bartonella henselae, *lymphadenitis*, *pediatrics*

## Abstract

**Background:** Prolonged fever for more than a week or fever of unknown origin in pediatric patients with or without soft tissue infection should raise suspicion for *Bartonella henselae* infection.

**Case Report:** A 10-year-old female presented to urgent care with a “bug bite” on the left ring finger, cough, and 2 to 3 days of low-grade fever. Ten days later, her symptoms progressed to soft tissue swelling of the left elbow without fracture on radiograph. Magnetic resonance imaging revealed multiple reniform masses with avid contrast enhancement consistent with suppurative adenitis. She was admitted for irrigation and debridement. The patient underwent surgical debridement with removal of infected lymph nodes. Histology revealed necrotizing granulomatous lymphadenitis. Polymerase chain reaction was positive for *B henselae*. Antibody titer revealed *B henselae* immunoglobulin G titer of 1:512 (reference, 1:64), and negative immunoglobulin M titers were indicative of mature immune response. The patient was treated with azithromycin 250 mg tablets twice daily for 3 days followed by 3 days of the 250 mg tablet once daily. Follow-up showed resolution of infection without symptoms concerning for visceral organ infection.

**Conclusion:** While the patient's initial presentation was vague, a complete history, quick follow-up, and decisive intervention prevented significant sequelae such as visceral organ involvement.

## INTRODUCTION

Cat scratch disease or lymphoreticulosis is a bacterial infection of the lymph nodes caused by *Bartonella henselae* and characterized by erythematous papules, prolonged fever, and progression to lymphadenitis in some cases. When cats scratch, lick, or bite, *B henselae* in the feline saliva can be transmitted through a break in the skin's integrity. Fleas transmit the pathogen to cats through biting, and the organism enters the feline saliva. However, *B henselae* has also been found in lice, soil, and detritus in many environments in the Americas,^1^ so *B henselae* can be the cause of soft tissue infection without the classic history of a cat bite or scratch, making it difficult to diagnose early.^2^ Definitive diagnosis is made with dissection and pathologic findings of pleomorphic gram-negative bacilli within the capillary walls of the infected lymph node and near areas of follicular hyperplasia or within microabscesses.^3^ Laboratory findings can aid in diagnosis through the use of antibody titers, immunoglobulin G (IgG) and immunoglobulin M (IgM), to test for active infection or exposure to *B henselae.*^4^ In the setting of symptoms that clinically correlate to cat scratch disease without lymphadenitis, elevated antibody titers are enough to prompt treatment with antibiotics; however, in the presence of abscess or lymph node infection, definitive treatment must include drainage or resection of the soft tissue infection. Samples of soft tissue should be sectioned for definitive diagnosis given a differential that includes mycobacterial infection and neoplasm.^4^ In children, *B henselae* can present with prolonged fever without soft tissue findings for weeks prior to symptoms of lymphadenitis, and titers should be considered in the workup for any pediatric patient with prolonged fever of unknown origin.^5^

## CASE REPORT

A 10-year-old female with a medical history significant for eczema presented to an urgent care clinic with the chief complaint of fever and cough for 2 to 3 days and a history of a “bug bite” on the ulnar aspect of the left ring finger 1 month prior. She endorsed purulent discharge from this sore; however, it was an erythematous papule without discharge at time of presentation. Her temperature was 100.8 °F. She denied shortness of breath, loss of taste and smell, headache, known sick contacts, gastrointestinal symptoms, sore throat, or ear pain. She was given ibuprofen and 2% mupirocin ointment for application to her “bug bite.”

**Figure 1.**
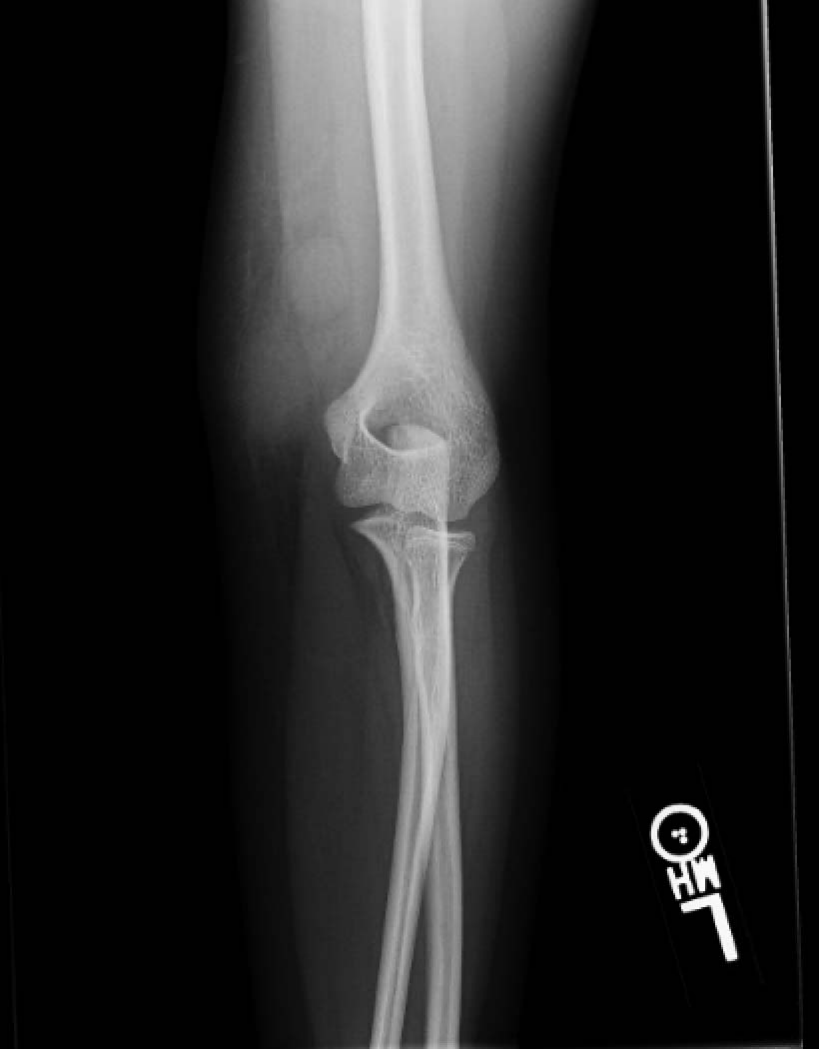
Radiograph of the left elbow shows no acute fracture process or significant edema.

Ten days later, she presented to the pediatric clinic with left medial elbow pain, swelling without erythema, tenderness to palpation, and continued subjective fever although she was afebrile at this visit. Her mother had noticed a firm, edematous mass over the left medial epicondyle 9 days after the initial presentation to urgent care. Left elbow radiograph revealed no acute fracture process or significant edema (Figure 1). The pediatrician was concerned for abscess. Because the patient was hemodynamically stable, the decision was made to refrain from antibiotics to ensure culture accuracy if she required surgery. Pediatric orthopedic surgery was consulted, and the patient was seen in a clinical setting the next day, 12 days after the initial presentation.

**Figure 2.**
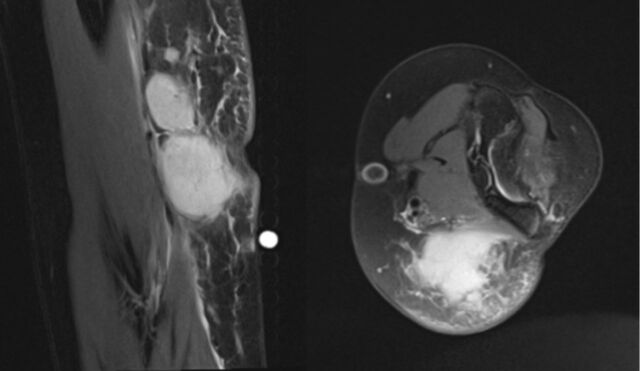
T2-weighted magnetic resonance imaging of the left elbow displays abscess formation.

Elevated erythrocyte sedimentation rate, C-reactive protein, and white blood cell count were concerning for infection (Table). Magnetic resonance imaging showed multiple reniform masses in the epitrochlear region with avid contrast enhancement. The largest mass measured 1.6 cm and demonstrated peripherally enhancing fluid consistent with suppurative adenitis and abscess formation (Figure 2). Associated abscess formation measured approximately 2.4 × 1.6 × 1.2 cm with surrounding soft tissue edema. Irrigation and debridement of the left elbow were recommended. Risks, benefits, and alternatives to the surgery were explained to the patient's mother, and informed consent was obtained. Thirteen days after her initial presentation to urgent care and 2 days after her presentation to the pediatrician, the patient was admitted and prepared for surgery.

**Table.** Laboratory Results Concerning for Infection

Test	Reference Range	Value
Erythrocyte sedimentation rate, mm/hr	0-36	41
C-reactive protein, mg/L	0-8.2	10.3
White blood cell count, WBC/μL	4,500-10,000	14,740

A medial approach to the elbow was used to access the abscess cavity. Purulent material was encountered as dissection was carried down through the subcutaneous tissues. Two infected lymph nodes were noted, excised, and sent for biopsy. Excisional debridement was carried out to the level of the fascia. The patient was discharged home the same day with 1-week follow-up in the orthopedic clinic scheduled for a wound check. The patient was prescribed azithromycin 250 mg twice daily for 3 days followed by 3 days of 250 mg once daily per the infectious disease service recommendations. Twelve days postoperatively, the patient returned for follow-up in clinic having completed the azithromycin with resolution of symptoms.

The patient's *B henselae* IgG titer returned a value of 1:512 (reference, 1:64). Her IgM titer was negative. Pathology reported the finding of necrotizing granulomatous lymphadenitis, and *Bartonella* polymerase chain reaction of the tissue was positive. The final diagnosis was infected left medial elbow lymph node/abscess caused by *B henselae*.

## DISCUSSION

Lymphadenitis, a frequent manifestation of cat scratch disease, presents a complex clinical scenario that demands swift recognition and appropriate intervention to avert potential complications. This case reports a distinctive clinical episode involving a 10-year-old female who developed medial epicondyle abscess and lymphadenitis with an insidious onset of symptoms in the setting of fever of unknown origin, ultimately necessitating surgical debridement and antibiotic therapy. This particular presentation deviates from the customary acute onset associated with cat scratch disease in patients with a compromised immune system or a self-resolving course in immunocompetent patients that does not result in end organ damage or disseminated infection.^6^ Typically, individuals with cat scratch disease manifest regional lymphadenopathy in the setting of feline contact. However, in this instance, the delayed progression of symptoms resulted in a presentation that can confuse clinicians, and a thorough history was required. This protracted course underscores the imperative for heightened clinical suspicion, as well as consideration of uncharacteristic presentations of cat scratch disease.

The untreated course of cat scratch disease, irrespective of its clinical presentation, can potentially culminate in disseminated disease affecting diverse organs.^6,7^ The etiologic agent, *B henselae*, has the capacity to infiltrate and persist within host cells, evading immune surveillance and inciting a systemic inflammatory response. When cat scratch disease remains untreated, it may progress to disseminated disease, involving organs such as the liver, spleen, bone marrow, and central nervous system.^7,8^ This progression may result in complications such as hepatosplenic involvement, neurologic symptoms, endocarditis, or severe systemic illness.^8-10^ Early diagnosis and intervention are imperative as untreated infection with *B henselae* has been known to progress to visceral organ involvement such as granulomatous hepatitis or granulomatous infection of the spleen with or without cutaneous manifestation.^11,12^

Once identification of *B henselae* is made, antibiotic treatment with azithromycin, or an equivalent therapy if azithromycin cannot be tolerated, can successfully decrease the swelling associated with lymphadenitis, decrease the potential for visceral organ involvement, and resolve infection.^13,14^ For patients who cannot tolerate azithromycin, oral clarithromycin 15 mg/kg twice daily for 7 to 10 days and oral trimethoprim-sulfamethoxazole 4 mg/kg daily for 7 to 10 days are acceptable alternatives.

## CONCLUSION

Fever of unknown origin in an otherwise healthy pediatric patient may be an early sign of cat scratch disease.

## Notes

*This article meets the Accreditation Council for Graduate Medical Education and the American Board of Medical Specialties Maintenance of Certification competencies for Patient Care and Medical Knowledge.*
